# Life-threatening hemobilia following plastic stent removal in a patient with biliary anastomotic stricture after liver transplantation

**DOI:** 10.1055/a-2742-5872

**Published:** 2025-12-04

**Authors:** Songming Ding, Hengkai Zhu, Li Zhuang, Shusen Zheng, Qiyong Li

**Affiliations:** 1 636046 Division of Hepatobiliary and Pancreatic Surgery, Shulan (Hangzhou) Hospital Affiliated to Zhejiang Shuren University, Shulan International Medical College, Hangzhou, China

Hemobilia, a term coined by Philip Sandblom in 1948 1 , refers to bleeding into the biliary system 2 , typically caused by iatrogenic injuries, accidental trauma, hepatobiliary malignancies, vascular malformations, and inflammatory or infectious diseases 3 . Hemobilia may be minor or major 4 . Here, we present a patient with an anastomotic stricture (AS) after liver transplantation (LT) who experienced fatal hemobilia while removing two plastic biliary stents (PBSs).

A 41-year-old male patient was on the waiting list for LT due to hepatitis B-related decompensated cirrhosis complicated with portal vein thrombosis and had a history of right hemicolectomy, cholecystectomy, and proper hepatic artery–portal vein fistula embolization ( Fig. 1 ). Two months post-LT, two PBSs were inserted through endoscopic retrograde cholangiography (ERC) due to the AS ( Fig. 2 **a, b** ). Three months later, the BPSs were exchanged ( Fig. 2 **c** ).

**Fig. 1:**
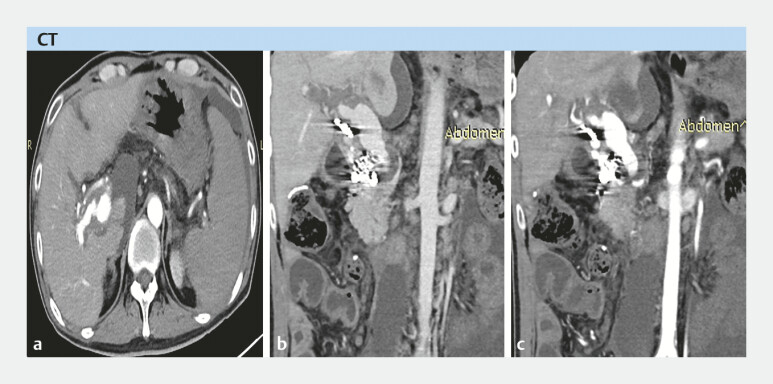
Enhanced computed tomography (CT) showing ( **a** ) liver cirrhosis with ascites and arterioportal fistula, ( **b** ) portal vein thrombosis with cavernous transformation, and ( **c** ) hepatic arterioportal fistula after embolization.

**Fig. 2:**
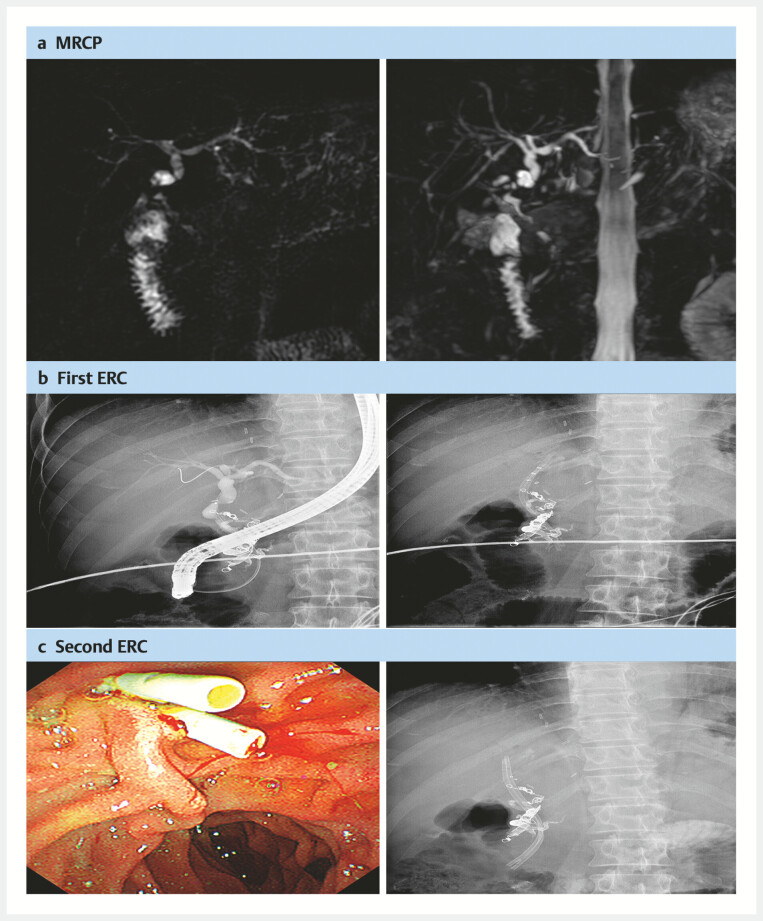
**a** Magnetic resonance cholangiopancreatography (MRCP) suggesting the biliary anastomotic stricture (AS) after liver transplantation (LT), **b** insertion of two plastic biliary stents (PBSs) using endoscopic retrograde cholangiography (ERC), and **c** reinsertion of two PBSs.

Nearly 7 months post-LT, the patient underwent a third ERC due to abdominal pain and elevated total bilirubin to 64 µmol/L, as well as imaging findings of bile duct gas accumulation and bile duct stones ( Fig. 3 ). During the process of removing one of the two previously inserted BPSs, copious fresh blood gushed out from the major papilla ( Fig. 4 ). After removing the two BPSs, biliary bleeding became uncontrollable ( Video 1 ). An emergent abdominal angiography revealed a communication between the the bile duct and superior mesenteric artery–portal vein fistula ( Video 1 ). Embolization was performed for the tortuous vessels originating from the superior mesenteric artery using gelatin sponge, iodized oil emulsion, and fibrin tissue adhesive emulsion ( Video 1 ). Finally, after extracting intraductal clots with a balloon catheter, a fully covered self-expanding metal stent (FCSEMS) was placed in the bile duct ( Video 1 ). Blood pressure once dropped to 60/37 mmHg, and hemoglobin dropped to 53 g/L. A total of 12 units of platelets, 3,100 ml of red blood cells, and 1330 ml of plasma were transfused. Subsequently, he was transferred to the intensive care unit for further treatment and discharged home on postoperative day 19. Ten days later, he was readmitted due to bloating and abnormal liver function. After splenic artery embolization, transjugular intrahepatic portosystemic shunt, and artificial liver treatment, liver function gradually improved. Twenty-six months post-LT, the patient’s liver function was almost normal, and FCSEMS remained in situ ( Fig. 5 ).

**Fig. 3:**
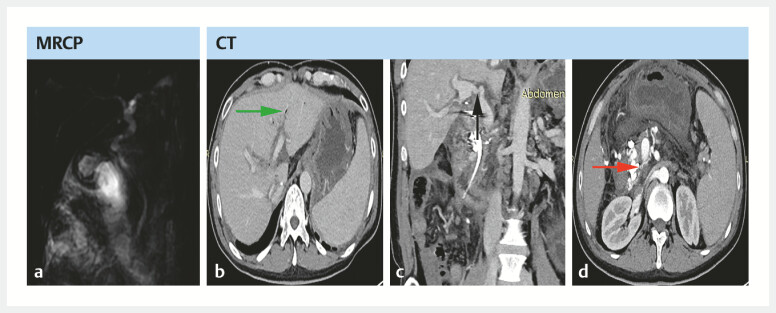
CT and MRCP before the third ERC. **a** MRCP showing bile duct stones and **b** CT showing bile duct gas accumulation (green arrow), **c** portal vein anastomotic stenosis (black arrow), and **d** peripancreatic arteriovenous fistula (red arrow). CT, computed tomography.

**Fig. 4:**
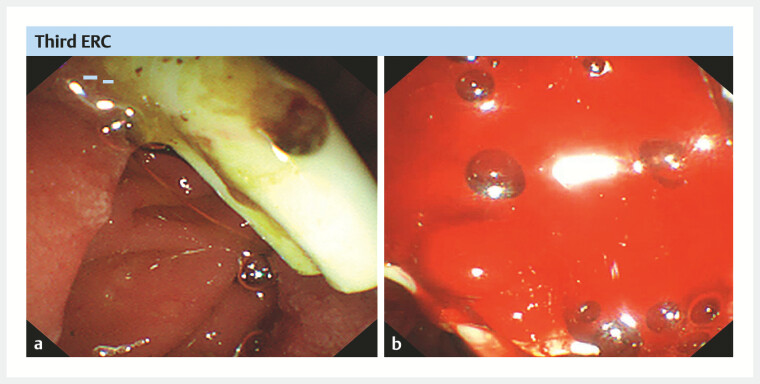
Major hemobilia following removal of previously inserted PBSs. **a** The PBSs inserted during the second ERC and **b** major hemobilia.

Endoscopic and radiological interventions for the life-threatening hemobilia.Video 1

**Fig. 5:**
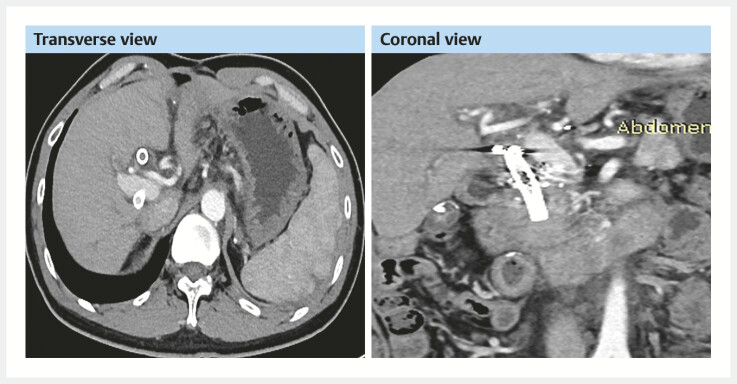
The fully covered self-expanding metal stent (FCSEMS) was still in place.

This patient’s hemobilia was related to chronic PBS-associated biliary inflammation 5 and peribiliary arteriovenous fistula. Continuing to remove plastic stents may be an aggravating factor when hemobilia was observed. Life-threatening hemobilia should be managed by ERC in conjunction with endovascular intervention, especially for patients with arteriovenous fistulas, in order to achieve hemostasis and maintain bile flow 3 .

Endoscopy_UCTN_Code_CPL_1AK_2AD

## Notes

Endoscopy E-Videos https://eref.thieme.de/e-videos
*E-Videos* is an open access online section of the journal *Endoscopy* , reporting on interesting cases and new techniques in gastroenterological endoscopy. All papers include a high-quality video and are published with a Creative Commons CC-BY license. Endoscopy E-Videos qualify for HINARI discounts and waivers and eligibility is automatically checked during the submission process. We grant 100% waivers to articles whose corresponding authors are based in Group A countries and 50% waivers to those who are based in Group B countries as classified by Research4Life (see: https://www.research4life.org/access/eligibility/ ). This section has its own submission website at https://mc.manuscriptcentral.com/e-videos .
